# *N*,*N*-Bis(4-nitro­phen­yl)acetamide

**DOI:** 10.1107/S1600536813005175

**Published:** 2013-02-28

**Authors:** Kokichi Nanaura, Tsunehisa Okuno

**Affiliations:** a Department of Material Science and Chemistry, Wakayama University, Sakaedani, Wakayama, 640-8510, Japan

## Abstract

In the title compound, C_14_H_11_N_3_O_5_, the dihedral angles between the amide group (r.m.s. deviation = 0.0429 Å) and the two benzene rings are 39.66 (6) and 63.04 (7)°. The dihedral angle between the benzene rings is 86.04 (7)°. The benzene rings form dihedral angles of 4.42 (5) and 8.91 (5)° with the adjacent nitro groups. In the crystal, mol­ecules are linked *via* a pair of C—H⋯O hydrogen bonds, forming inversion dimers, which are linked *via* a second pair of C—H⋯O hydrogen bonds, forming chains propagating along [100].

## Related literature

For the related structures of diphenyl­acetamide derivatives, see: Kim *et al.* (2003 ▶); Krigbaum *et al.* (1968 ▶); Yamasaki *et al.* (2003 ▶).
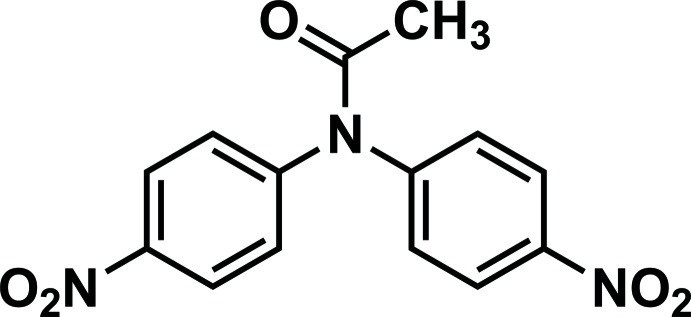


## Experimental

### 

#### Crystal data

C_14_H_11_N_3_O_5_
*M* *_r_* = 301.26
Triclinic, 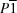

*a* = 7.454 (3) Å
*b* = 8.070 (4) Å
*c* = 12.078 (5) Å
α = 81.449 (10)°
β = 74.676 (10)°
γ = 88.062 (13)°
*V* = 692.9 (6) Å^3^
*Z* = 2
Mo *K*α radiation
μ = 0.11 mm^−1^
*T* = 93 K
0.10 × 0.10 × 0.08 mm

#### Data collection

Rigaku Saturn724+ diffractometer
Absorption correction: numerical (*NUMABS*; Rigaku, 1999 ▶) *T*_min_ = 0.984, *T*_max_ = 0.991
4684 measured reflections
2397 independent reflections
2038 reflections with *F* ^2^ > 2σ(*F* ^2^)
*R*_int_ = 0.096

#### Refinement

*R*[*F* ^2^ > 2σ(*F* ^2^)] = 0.053
*wR*(*F* ^2^) = 0.138
*S* = 1.06
2397 reflections
200 parameters
H-atom parameters constrained
Δρ_max_ = 0.27 e Å^−3^
Δρ_min_ = −0.27 e Å^−3^

### 

Data collection: *CrystalClear* (Rigaku, 2008 ▶); cell refinement: *CrystalClear*; data reduction: *CrystalClear*; program(s) used to solve structure: *SHELXD* (Schneider, *et al.*, 2002 ▶); program(s) used to refine structure: *SHELXL97* (Sheldrick, 2008 ▶); molecular graphics: *ORTEP-3 for Windows* (Farrugia, 2012 ▶); software used to prepare material for publication: *CrystalStructure* (Rigaku, 2010 ▶).

## Supplementary Material

Crystal structure: contains datablock(s) global, I. DOI: 10.1107/S1600536813005175/ff2098sup1.cif

Structure factors: contains datablock(s) I. DOI: 10.1107/S1600536813005175/ff2098Isup2.hkl

Supplementary material file. DOI: 10.1107/S1600536813005175/ff2098Isup3.cml

Additional supplementary materials:  crystallographic information; 3D view; checkCIF report

## Figures and Tables

**Table 1:** Hydrogen-bond geometry (Å, °)

*D*—H⋯*A*	*D*—H	H⋯*A*	*D*⋯*A*	*D*—H⋯*A*
C3—H3⋯O5^i^	0.95	2.39	3.183 (3)	141
C8—H8⋯O5^ii^	0.95	2.34	3.204 (3)	152

Symmetry codes: (i) 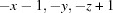; (ii) 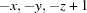.

**Fig. 1.**
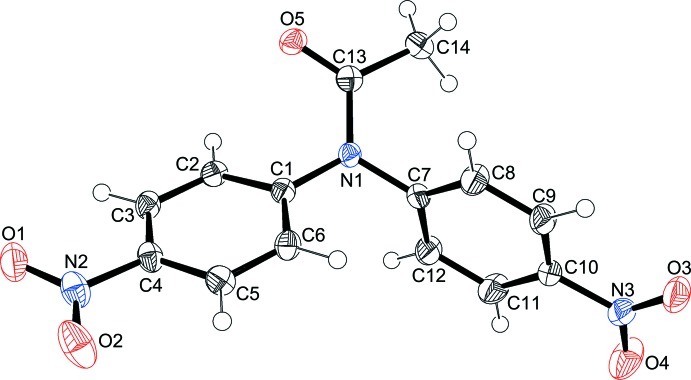
The asymmetric unit of the title compound with atom-numbering scheme. Displacement ellipsoids are drawn at the 50% probability level and H atoms are shown as small spheres.

**Fig. 2.**
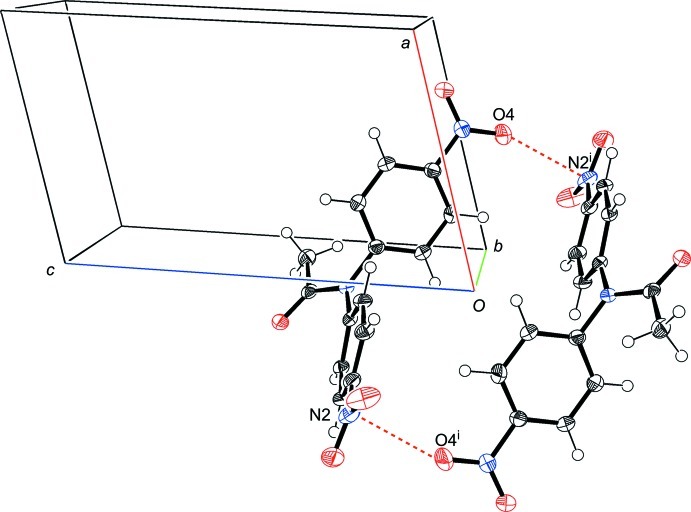
A view of the intermolecular interactions in the title compound. [Symmetry code: (i) -x, -y, -z.]

## supplementary crystallographic information

### Comment

The title compound, C_14_H_11_N_3_O_5_, is a derivative of diphenylacetamides, whose structures are paid attention because of relationship between their structures and molecular recognition functions (Yamasaki *et al.*, 2003).

The structure of the C1/C7/N1/C13/O5/C14 amide group is almost planar (r.m.s. deviation = 0.0429 Å) The dihedral angles of the plane of the amide group with the C1—C6 phenyl ring and the C7—C12 phenyl ring are 39.66 (6)° and 63.04 (7)°, respectively. The dihedral angle between the two phenyl rings is 86.04 (7)°. In diphenylacetamide derivatives, electron deficient aromatic rings have a tendency to make a smaller dihedral angle to an amide part (Kim *et al.*, 2003; Krigbaum *et al.*, 1968; Yamasaki *et al.*, 2003). The obtained geometry has a good agreement with the tendency.

The dihedral angle of the N2/O1/O2 nitro group with the C1—C6 phenyl ring are 8.91 (5)°. The dihedral angle of the N3/O3/O4 nitro group with the C7—C12 phenyl ring is 4.42 (5)°. The nitro groups have good conjugations with the corresponding phenyl groups. Intermolecular contacts are observed beween the nitro groups related by an inversion symmetry, where O4···N2^i^ and N2···O4^i^ are 2.948 (3) Å [Symmetry code: (i) -*x*, -*y*, -*z*].

### Experimental

Single crystals with sufficient quality for X-ray crystallographical analysis were prepared by recrystallization from a mixed solution of dichloromethane and hexane.

### Refinement

The C-bound H atoms were placed at ideal positions and were refined as riding on their parent C atoms. *U*_iso_(H) values of the H atoms were set at 1.2*U*_eq_(parent atom).

### Crystal data

**Table:**

C_14_H_11_N_3_O_5_	*Z* = 2
*M**_r_* = 301.26	*F*(000) = 312.00
Triclinic, *P*1	*D*_x_ = 1.444 Mg m^−^^3^
Hall symbol: -P 1	Mo *K*α radiation, λ = 0.71075 Å
*a* = 7.454 (3) Å	Cell parameters from 1648 reflections
*b* = 8.070 (4) Å	θ = 1.8–25.0°
*c* = 12.078 (5) Å	µ = 0.11 mm^−^^1^
α = 81.449 (10)°	*T* = 93 K
β = 74.676 (10)°	Platelet, colourless
γ = 88.062 (13)°	0.10 × 0.10 × 0.08 mm
*V* = 692.9 (6) Å^3^	

### Data collection

**Table:**

Rigaku Saturn724+ diffractometer	2038 reflections with *F*^2^ > 2σ(*F*^2^)
Detector resolution: 28.445 pixels mm^-1^	*R*_int_ = 0.096
ω scans	θ_max_ = 25.0°
Absorption correction: numerical (*NUMABS*; Rigaku, 1999)	*h* = −8→8
*T*_min_ = 0.984, *T*_max_ = 0.991	*k* = −9→9
4684 measured reflections	*l* = −14→14
2397 independent reflections	

### Refinement

**Table:**

Refinement on *F*^2^	Secondary atom site location: difference Fourier map
*R*[*F*^2^ > 2σ(*F*^2^)] = 0.053	Hydrogen site location: inferred from neighbouring sites
*wR*(*F*^2^) = 0.138	H-atom parameters constrained
*S* = 1.06	*w* = 1/[σ^2^(*F*_o_^2^) + (0.0625*P*)^2^ + 0.2501*P*] where *P* = (*F*_o_^2^ + 2*F*_c_^2^)/3
2397 reflections	(Δ/σ)_max_ < 0.001
200 parameters	Δρ_max_ = 0.27 e Å^−^^3^
0 restraints	Δρ_min_ = −0.27 e Å^−^^3^
Primary atom site location: structure-invariant direct methods	

### Special details

**Table:**

Refinement. Refinement was performed using all reflections. The weighted *R*-factor (*wR*) and goodness of fit (*S*) are based on *F*^2^. *R*-factor (gt) are based on *F*. The threshold expression of *F*^2^ > 2.0 σ(*F*^2^) is used only for calculating *R*-factor (gt).

### Fractional atomic coordinates and isotropic or equivalent isotropic displacement parameters (Å^2^)

**Table:**

	*x*	*y*	*z*	*U*_iso_*/*U*_eq_	
O1	−0.5557 (2)	−0.5404 (2)	0.37979 (13)	0.0390 (4)	
O2	−0.3035 (2)	−0.6254 (2)	0.26745 (17)	0.0472 (5)	
O3	0.74805 (19)	0.2913 (3)	−0.02433 (13)	0.0439 (5)	
O4	0.5763 (3)	0.3680 (3)	−0.13945 (14)	0.0527 (6)	
O5	−0.19693 (17)	0.09710 (17)	0.50403 (11)	0.0252 (4)	
N1	−0.03594 (19)	0.0866 (2)	0.31681 (13)	0.0197 (4)	
N2	−0.3953 (3)	−0.5182 (3)	0.31981 (16)	0.0303 (5)	
N3	0.5958 (3)	0.3042 (3)	−0.04519 (14)	0.0297 (5)	
C1	−0.1318 (3)	−0.0612 (3)	0.31114 (15)	0.0202 (4)	
C2	−0.3232 (3)	−0.0792 (3)	0.35967 (16)	0.0220 (5)	
C3	−0.4102 (3)	−0.2286 (3)	0.36144 (16)	0.0234 (5)	
C4	−0.3058 (3)	−0.3557 (3)	0.31174 (16)	0.0235 (5)	
C5	−0.1181 (3)	−0.3371 (3)	0.25680 (17)	0.0252 (5)	
C6	−0.0311 (3)	−0.1892 (3)	0.25763 (17)	0.0247 (5)	
C7	0.1247 (3)	0.1395 (3)	0.22310 (16)	0.0214 (5)	
C8	0.3012 (3)	0.1129 (3)	0.24046 (17)	0.0300 (5)	
C9	0.4555 (3)	0.1664 (3)	0.15180 (17)	0.0307 (5)	
C10	0.4306 (3)	0.2425 (3)	0.04780 (16)	0.0238 (5)	
C11	0.2570 (3)	0.2667 (3)	0.02760 (17)	0.0330 (5)	
C12	0.1024 (3)	0.2137 (3)	0.11739 (17)	0.0299 (5)	
C13	−0.0767 (3)	0.1569 (3)	0.41913 (16)	0.0213 (5)	
C14	0.0321 (3)	0.3092 (3)	0.42132 (18)	0.0290 (5)	
H2	−0.3936	0.0110	0.3914	0.0264*	
H3	−0.5398	−0.2433	0.3963	0.0280*	
H5	−0.0506	−0.4242	0.2193	0.0302*	
H6	0.0982	−0.1747	0.2215	0.0297*	
H8	0.3154	0.0582	0.3129	0.0360*	
H9	0.5769	0.1508	0.1627	0.0368*	
H11	0.2438	0.3183	−0.0458	0.0396*	
H12	−0.0188	0.2285	0.1060	0.0358*	
H14A	0.0786	0.3679	0.3422	0.0347*	
H14B	0.1373	0.2749	0.4538	0.0347*	
H14C	−0.0486	0.3841	0.4695	0.0347*	

### Atomic displacement parameters (Å^2^)

**Table:**

	*U*^11^	*U*^22^	*U*^33^	*U*^12^	*U*^13^	*U*^23^
O1	0.0354 (9)	0.0405 (10)	0.0406 (9)	−0.0158 (7)	−0.0123 (7)	0.0037 (8)
O2	0.0371 (9)	0.0279 (9)	0.0846 (14)	0.0051 (7)	−0.0246 (9)	−0.0190 (9)
O3	0.0219 (8)	0.0775 (14)	0.0301 (9)	−0.0079 (8)	−0.0056 (7)	−0.0009 (9)
O4	0.0369 (9)	0.0839 (15)	0.0282 (9)	−0.0039 (9)	−0.0072 (7)	0.0190 (9)
O5	0.0200 (7)	0.0326 (8)	0.0224 (7)	0.0023 (6)	−0.0038 (6)	−0.0061 (6)
N1	0.0151 (8)	0.0236 (9)	0.0205 (8)	−0.0001 (6)	−0.0051 (6)	−0.0026 (7)
N2	0.0272 (10)	0.0289 (10)	0.0390 (10)	−0.0025 (8)	−0.0184 (8)	−0.0001 (8)
N3	0.0267 (9)	0.0390 (11)	0.0221 (9)	−0.0034 (8)	−0.0047 (7)	−0.0027 (8)
C1	0.0174 (9)	0.0244 (10)	0.0198 (9)	0.0003 (8)	−0.0080 (7)	−0.0006 (8)
C2	0.0171 (9)	0.0281 (11)	0.0215 (9)	0.0042 (8)	−0.0066 (8)	−0.0037 (8)
C3	0.0158 (9)	0.0306 (11)	0.0237 (10)	−0.0009 (8)	−0.0067 (8)	−0.0008 (9)
C4	0.0221 (10)	0.0233 (11)	0.0266 (10)	−0.0025 (8)	−0.0118 (8)	0.0017 (8)
C5	0.0213 (10)	0.0262 (11)	0.0299 (11)	0.0040 (8)	−0.0087 (8)	−0.0073 (9)
C6	0.0154 (9)	0.0303 (11)	0.0283 (10)	0.0016 (8)	−0.0051 (8)	−0.0052 (9)
C7	0.0186 (9)	0.0236 (10)	0.0227 (10)	−0.0017 (8)	−0.0056 (8)	−0.0050 (8)
C8	0.0226 (10)	0.0429 (13)	0.0232 (10)	−0.0003 (9)	−0.0080 (8)	0.0034 (9)
C9	0.0179 (10)	0.0480 (14)	0.0268 (11)	−0.0016 (9)	−0.0083 (8)	−0.0025 (10)
C10	0.0198 (10)	0.0280 (11)	0.0228 (10)	−0.0026 (8)	−0.0034 (8)	−0.0048 (9)
C11	0.0289 (11)	0.0468 (14)	0.0220 (10)	0.0019 (10)	−0.0092 (9)	0.0032 (10)
C12	0.0192 (10)	0.0457 (13)	0.0257 (10)	−0.0003 (9)	−0.0093 (8)	−0.0021 (10)
C13	0.0167 (9)	0.0247 (11)	0.0243 (10)	0.0062 (8)	−0.0089 (8)	−0.0039 (8)
C14	0.0300 (11)	0.0314 (12)	0.0279 (11)	−0.0031 (9)	−0.0104 (9)	−0.0062 (9)

### Geometric parameters (Å, º)

**Table:**

O1—N2	1.226 (3)	C7—C12	1.376 (3)
O2—N2	1.229 (3)	C8—C9	1.379 (3)
O3—N3	1.224 (3)	C9—C10	1.369 (3)
O4—N3	1.221 (3)	C10—C11	1.382 (3)
O5—C13	1.218 (2)	C11—C12	1.386 (3)
N1—C1	1.430 (3)	C13—C14	1.502 (3)
N1—C7	1.439 (2)	C2—H2	0.950
N1—C13	1.393 (3)	C3—H3	0.950
N2—C4	1.470 (3)	C5—H5	0.950
N3—C10	1.473 (3)	C6—H6	0.950
C1—C2	1.395 (3)	C8—H8	0.950
C1—C6	1.392 (3)	C9—H9	0.950
C2—C3	1.383 (3)	C11—H11	0.950
C3—C4	1.379 (3)	C12—H12	0.950
C4—C5	1.384 (3)	C14—H14A	0.980
C5—C6	1.380 (3)	C14—H14B	0.980
C7—C8	1.391 (3)	C14—H14C	0.980
			
O1···C3	2.730 (3)	H11···H12	2.3500
O1···C5	3.552 (3)	H12···H14A	3.4692
O2···C3	3.551 (3)	O1···H3^i^	3.2240
O2···C5	2.716 (3)	O1···H14A^iii^	3.0122
O3···C9	2.712 (3)	O1···H14B^iii^	2.6480
O3···C11	3.554 (3)	O1···H14C^iii^	3.5911
O4···C9	3.543 (3)	O1···H14C^ix^	3.3464
O4···C11	2.740 (3)	O2···H2^iv^	3.0985
O5···C1	2.746 (3)	O2···H9^iii^	2.6542
O5···C2	2.765 (3)	O2···H11^ii^	3.3131
O5···C7	3.576 (3)	O2···H12^iv^	2.8331
C1···C4	2.744 (3)	O2···H14A^iv^	3.2039
C1···C8	3.408 (3)	O2···H14C^iv^	3.4762
C1···C12	3.163 (3)	O3···H5^v^	2.8961
C2···C5	2.786 (3)	O3···H6^v^	2.6499
C2···C13	2.977 (3)	O3···H11^vi^	3.3887
C3···C6	2.780 (3)	O3···H12^vii^	2.6276
C6···C7	2.860 (3)	O4···H3^ii^	3.4790
C6···C8	3.480 (4)	O4···H5^v^	3.4369
C6···C12	3.481 (4)	O4···H6^v^	2.8701
C6···C13	3.595 (4)	O4···H14A^vi^	3.5560
C7···C10	2.725 (3)	O5···H2^ix^	3.0802
C7···C14	2.850 (4)	O5···H3^ix^	2.3895
C8···C11	2.780 (3)	O5···H8^viii^	2.3362
C8···C13	3.109 (3)	O5···H14B^viii^	3.0071
C8···C14	3.131 (3)	N2···H3^i^	3.5938
C9···C12	2.776 (3)	N2···H9^iii^	3.5400
C12···C13	3.500 (3)	N2···H11^ii^	3.3749
O1···O1^i^	3.380 (3)	N2···H14C^iv^	3.5320
O1···O4^ii^	3.067 (3)	N3···H5^v^	3.5638
O1···C3^i^	3.345 (3)	N3···H6^v^	2.9605
O1···C4^i^	3.570 (3)	C1···H9^xii^	3.4281
O1···C14^iii^	3.228 (3)	C1···H14B^viii^	3.0862
O2···O4^ii^	3.355 (3)	C2···H2^ix^	3.2949
O2···N1^iv^	3.105 (3)	C2···H8^xii^	3.0367
O2···C9^iii^	3.199 (4)	C2···H9^xii^	3.0316
O2···C12^iv^	3.415 (3)	C2···H14B^viii^	3.1521
O2···C13^iv^	3.128 (3)	C3···H8^xii^	3.1294
O2···C14^iv^	3.474 (4)	C3···H14B^viii^	3.3704
O3···C5^v^	3.361 (3)	C4···H11^ii^	3.0913
O3···C6^v^	3.240 (3)	C4···H14B^viii^	3.5370
O3···C11^vi^	3.560 (4)	C4···H14C^iv^	3.4905
O3···C12^vii^	3.501 (3)	C5···H11^ii^	2.9211
O4···O1^ii^	3.067 (3)	C5···H14A^iv^	2.9518
O4···O2^ii^	3.355 (3)	C5···H14B^viii^	3.5699
O4···N2^ii^	2.948 (3)	C5···H14C^iv^	3.2916
O4···C3^ii^	3.568 (4)	C6···H14B^viii^	3.3341
O4···C4^ii^	3.270 (3)	C8···H2^vii^	3.2859
O4···C10^vi^	3.479 (4)	C8···H3^vii^	3.5429
O5···O5^viii^	3.266 (3)	C9···H2^vii^	3.4298
O5···N1^viii^	3.279 (3)	C12···H5^x^	3.3853
O5···C2^ix^	3.518 (3)	C13···H3^ix^	3.2539
O5···C3^ix^	3.183 (3)	C13···H8^viii^	3.4855
O5···C8^viii^	3.204 (3)	C13···H14B^viii^	3.5847
O5···C13^viii^	3.062 (3)	C14···H5^x^	3.1790
O5···C14^viii^	3.536 (3)	C14···H14C^xiii^	2.9936
N1···O2^x^	3.105 (3)	H2···O2^x^	3.0985
N1···O5^viii^	3.279 (3)	H2···O5^ix^	3.0802
N2···O4^ii^	2.948 (3)	H2···C2^ix^	3.2949
C2···O5^ix^	3.518 (3)	H2···C8^xii^	3.2859
C3···O1^i^	3.345 (3)	H2···C9^xii^	3.4298
C3···O4^ii^	3.568 (4)	H2···H2^ix^	2.6662
C3···O5^ix^	3.183 (3)	H2···H3^ix^	3.3208
C4···O1^i^	3.570 (3)	H2···H8^xii^	2.5836
C4···O4^ii^	3.270 (3)	H2···H9^xii^	2.8867
C5···O3^v^	3.361 (3)	H2···H14B^viii^	3.5740
C5···C14^iv^	3.553 (3)	H3···O1^i^	3.2240
C6···O3^v^	3.240 (3)	H3···O4^ii^	3.4790
C8···O5^viii^	3.204 (3)	H3···O5^ix^	2.3895
C9···O2^xi^	3.199 (4)	H3···N2^i^	3.5938
C10···O4^vi^	3.479 (4)	H3···C8^xii^	3.5429
C11···O3^vi^	3.560 (4)	H3···C13^ix^	3.2539
C12···O2^x^	3.415 (3)	H3···H2^ix^	3.3208
C12···O3^xii^	3.501 (3)	H3···H8^xii^	2.7875
C13···O2^x^	3.128 (3)	H3···H14C^ix^	3.2232
C13···O5^viii^	3.062 (3)	H5···O3^v^	2.8961
C13···C13^viii^	3.324 (3)	H5···O4^v^	3.4369
C14···O1^xi^	3.228 (3)	H5···N3^v^	3.5638
C14···O2^x^	3.474 (4)	H5···C12^iv^	3.3853
C14···O5^viii^	3.536 (3)	H5···C14^iv^	3.1790
C14···C5^x^	3.553 (3)	H5···H11^ii^	2.8595
O1···H3	2.4445	H5···H12^iv^	3.2729
O2···H5	2.4240	H5···H14A^iv^	2.4184
O3···H9	2.4141	H5···H14C^iv^	3.1864
O4···H11	2.4557	H6···O3^v^	2.6499
O5···H2	2.4258	H6···O4^v^	2.8701
O5···H14A	3.1191	H6···N3^v^	2.9605
O5···H14B	2.7956	H8···O5^viii^	2.3362
O5···H14C	2.5300	H8···C2^vii^	3.0367
N1···H2	2.6396	H8···C3^vii^	3.1294
N1···H6	2.5961	H8···C13^viii^	3.4855
N1···H8	2.6096	H8···H2^vii^	2.5836
N1···H12	2.6044	H8···H3^vii^	2.7875
N1···H14A	2.5407	H9···O2^xi^	2.6542
N1···H14B	2.9533	H9···N2^xi^	3.5400
N1···H14C	3.2219	H9···C1^vii^	3.4281
N2···H3	2.6186	H9···C2^vii^	3.0316
N2···H5	2.6197	H9···H2^vii^	2.8867
N3···H9	2.5992	H9···H12^vii^	2.9759
N3···H11	2.6241	H11···O2^ii^	3.3131
C1···H3	3.2660	H11···O3^vi^	3.3887
C1···H5	3.2663	H11···N2^ii^	3.3749
C1···H8	3.5073	H11···C4^ii^	3.0913
C1···H12	3.1019	H11···C5^ii^	2.9211
C2···H6	3.2698	H11···H5^ii^	2.8595
C3···H5	3.2729	H12···O2^x^	2.8331
C4···H2	3.2393	H12···O3^xii^	2.6276
C4···H6	3.2372	H12···H5^x^	3.2729
C5···H3	3.2729	H12···H9^xii^	2.9759
C6···H2	3.2704	H14A···O1^xi^	3.0122
C6···H8	3.5719	H14A···O2^x^	3.2039
C6···H12	3.5807	H14A···O4^vi^	3.5560
C7···H6	2.5530	H14A···C5^x^	2.9518
C7···H9	3.2561	H14A···H5^x^	2.4184
C7···H11	3.2541	H14A···H14C^xiii^	3.2074
C7···H14A	2.4635	H14B···O1^xi^	2.6480
C7···H14B	3.1646	H14B···O5^viii^	3.0071
C8···H6	2.8821	H14B···C1^viii^	3.0862
C8···H12	3.2625	H14B···C2^viii^	3.1521
C8···H14A	2.8181	H14B···C3^viii^	3.3704
C8···H14B	3.0116	H14B···C4^viii^	3.5370
C9···H11	3.2675	H14B···C5^viii^	3.5699
C10···H8	3.2278	H14B···C6^viii^	3.3341
C10···H12	3.2382	H14B···C13^viii^	3.5847
C11···H9	3.2672	H14B···H2^viii^	3.5740
C12···H6	3.1965	H14B···H14C^xiii^	3.0306
C12···H8	3.2615	H14C···O1^xi^	3.5911
C12···H14A	3.1125	H14C···O1^ix^	3.3464
C13···H2	2.7932	H14C···O2^x^	3.4762
C13···H8	2.9975	H14C···N2^x^	3.5320
C14···H8	3.0518	H14C···C4^x^	3.4905
H2···H3	2.3419	H14C···C5^x^	3.2916
H5···H6	2.3380	H14C···C14^xiii^	2.9936
H6···H8	3.0232	H14C···H3^ix^	3.2232
H6···H12	3.5205	H14C···H5^x^	3.1864
H8···H9	2.3401	H14C···H14A^xiii^	3.2074
H8···H14A	3.0202	H14C···H14B^xiii^	3.0306
H8···H14B	2.6923	H14C···H14C^xiii^	2.3144
			
C1—N1—C7	117.73 (16)	C10—C11—C12	118.19 (19)
C1—N1—C13	120.10 (14)	C7—C12—C11	119.90 (19)
C7—N1—C13	121.12 (17)	O5—C13—N1	121.08 (18)
O1—N2—O2	123.71 (19)	O5—C13—C14	121.06 (19)
O1—N2—C4	118.39 (17)	N1—C13—C14	117.86 (15)
O2—N2—C4	117.88 (16)	C1—C2—H2	120.083
O3—N3—O4	122.36 (16)	C3—C2—H2	120.077
O3—N3—C10	118.43 (17)	C2—C3—H3	120.539
O4—N3—C10	119.20 (17)	C4—C3—H3	120.544
N1—C1—C2	120.95 (17)	C4—C5—H5	120.727
N1—C1—C6	119.08 (15)	C6—C5—H5	120.731
C2—C1—C6	119.96 (19)	C1—C6—H6	119.844
C1—C2—C3	119.84 (18)	C5—C6—H6	119.836
C2—C3—C4	118.92 (16)	C7—C8—H8	120.234
N2—C4—C3	119.07 (16)	C9—C8—H8	120.228
N2—C4—C5	118.67 (17)	C8—C9—H9	120.571
C3—C4—C5	122.24 (19)	C10—C9—H9	120.567
C4—C5—C6	118.54 (18)	C10—C11—H11	120.906
C1—C6—C5	120.32 (16)	C12—C11—H11	120.905
N1—C7—C8	119.28 (17)	C7—C12—H12	120.047
N1—C7—C12	119.89 (17)	C11—C12—H12	120.052
C8—C7—C12	120.83 (16)	C13—C14—H14A	109.475
C7—C8—C9	119.54 (19)	C13—C14—H14B	109.471
C8—C9—C10	118.86 (19)	C13—C14—H14C	109.473
N3—C10—C9	118.49 (17)	H14A—C14—H14B	109.467
N3—C10—C11	118.85 (17)	H14A—C14—H14C	109.475
C9—C10—C11	122.65 (16)	H14B—C14—H14C	109.467
			
C1—N1—C7—C8	−104.8 (2)	N1—C1—C2—C3	−174.74 (15)
C1—N1—C7—C12	74.4 (3)	N1—C1—C6—C5	176.24 (15)
C7—N1—C1—C2	−148.93 (15)	C2—C1—C6—C5	−2.9 (3)
C7—N1—C1—C6	32.0 (3)	C6—C1—C2—C3	4.4 (3)
C1—N1—C13—O5	−1.9 (3)	C1—C2—C3—C4	−1.9 (3)
C1—N1—C13—C14	178.27 (14)	C2—C3—C4—N2	176.24 (16)
C13—N1—C1—C2	42.7 (3)	C2—C3—C4—C5	−2.1 (3)
C13—N1—C1—C6	−136.44 (16)	N2—C4—C5—C6	−174.77 (16)
C7—N1—C13—O5	−169.91 (16)	C3—C4—C5—C6	3.6 (3)
C7—N1—C13—C14	10.3 (3)	C4—C5—C6—C1	−1.0 (3)
C13—N1—C7—C8	63.5 (3)	N1—C7—C8—C9	−178.66 (17)
C13—N1—C7—C12	−117.3 (2)	N1—C7—C12—C11	179.13 (17)
O1—N2—C4—C3	−6.7 (3)	C8—C7—C12—C11	−1.7 (4)
O1—N2—C4—C5	171.77 (17)	C12—C7—C8—C9	2.1 (4)
O2—N2—C4—C3	174.59 (18)	C7—C8—C9—C10	−1.0 (4)
O2—N2—C4—C5	−7.0 (3)	C8—C9—C10—N3	178.05 (19)
O3—N3—C10—C9	−3.0 (3)	C8—C9—C10—C11	−0.5 (4)
O3—N3—C10—C11	175.68 (18)	N3—C10—C11—C12	−177.59 (18)
O4—N3—C10—C9	177.92 (19)	C9—C10—C11—C12	1.0 (4)
O4—N3—C10—C11	−3.4 (3)	C10—C11—C12—C7	0.1 (4)

Symmetry codes: (i) −*x*−1, −*y*−1, −*z*+1; (ii) −*x*, −*y*, −*z*; (iii) *x*−1, *y*−1, *z*; (iv) *x*, *y*−1, *z*; (v) −*x*+1, −*y*, −*z*; (vi) −*x*+1, −*y*+1, −*z*; (vii) *x*+1, *y*, *z*; (viii) −*x*, −*y*, −*z*+1; (ix) −*x*−1, −*y*, −*z*+1; (x) *x*, *y*+1, *z*; (xi) *x*+1, *y*+1, *z*; (xii) *x*−1, *y*, *z*; (xiii) −*x*, −*y*+1, −*z*+1.

### Hydrogen-bond geometry (Å, º)

**Table:**

*D*—H···*A*	*D*—H	H···*A*	*D*···*A*	*D*—H···*A*
C3—H3···O5^ix^	0.95	2.39	3.183 (3)	141
C8—H8···O5^viii^	0.95	2.34	3.204 (3)	152

Symmetry codes: (viii) −*x*, −*y*, −*z*+1; (ix) −*x*−1, −*y*, −*z*+1.
